# Identification and Characterization of the Chitin Synthase Genes From the Fish Pathogen *Saprolegnia parasitica*

**DOI:** 10.3389/fmicb.2019.02873

**Published:** 2019-12-13

**Authors:** Elzbieta Rzeszutek, Sara M. Díaz-Moreno, Vincent Bulone

**Affiliations:** ^1^Division of Glycoscience, Department of Chemistry, School of Engineering Sciences in Chemistry, Biotechnology and Health, KTH Royal Institute of Technology, AlbaNova University Centre, Stockholm, Sweden; ^2^ARC Centre of Excellence in Plant Cell Walls, School of Agriculture, Food and Wine, The University of Adelaide, Urrbrae, SA, Australia

**Keywords:** chitin synthase genes, chitin biosynthesis, nikkomycin Z, oomycetes, *Saprolegnia parasitica*

## Abstract

*Saprolegnia parasitica* is a pathogenic oomycete responsible for severe fish infections. Despite its low abundance in the cell wall of *S. parasitica*, chitin is essential for hyphal growth as the inhibition of its biosynthesis leads to highly reduced growth. Here we identified and characterized chitin synthases (CHS) from *S. parasitica* as potential targets for anti-oomycete drugs. Bioinformatics analyses allowed the identification of six different putative *Chs* genes in the genome of the pathogen. The total number of genes was confirmed by Southern blot analysis and their expression levels were determined by quantitative PCR. Four of the six *Chs* genes were expressed in the mycelium, while the two others exhibited undetectable levels of expression. The mycelium was highly sensitive to the addition of nikkomycin Z (NZ) in the culture medium, which led to a decreased amount of chitin in the cell wall by up to 40% in the conditions tested, and to the formation of abnormal branching structures in the hyphae. The presence of NZ increased the expression level of one of the genes, *Chs3*, suggesting that the corresponding product is compensating the disruption of chitin biosynthesis in the hyphae. In addition, the activity of isolated CHS was strongly inhibited by NZ *in vitro*. Altogether our data indicate the importance of CHS for the vegetative growth of *S. parasitica* and demonstrate that these enzymes represent promising targets for the control of diseases caused by oomycetes.

## Introduction

The oomycete lineage includes highly destructive plant and animal pathogens (Latijnhouwers et al., 2003; Phillips et al., 2008). Oomycetes are currently classified in the heterokont phylum and, despite their fungal-like morphology, they are more closely related to diatoms and brown algae than to fungi (Beakes et al., 2012). The species *Saprolegnia parasitica* is ubiquitous to all freshwater environments, and it infects wild and cultivated fish and crustaceans, causing the disease saprolegniosis. This infection currently represents a severe threat to the aquaculture industry and also to some wild populations of animals (Derevnina et al., 2016). Until 2002, the compound malachite green was used in aquaculture to keep *S. parasitica* growth and infection under control, but this chemical was internationally banned due to its carcinogenic and toxicological effects. As a direct consequence, saprolegniosis has experienced a resurgence and it currently represents one of the main threats to aquaculture (van West, 2006). It primarily affects the farming of salmonid species where it causes losses of tens of millions of euros per year in the major producing countries. In the United States, *S. parasitica* kills catfish causing financial losses of up to 50% (van West, 2006). At present, there are no efficient and environmentally friendly disease control methods available against this pathogen, hence the urgent need to develop new management strategies.

The cell wall is a promising target for anti-oomycete compounds as it provides a vital protective barrier to the microorganisms and is involved in many essential biological processes including growth, cell division, signaling, and interactions with the environment (Georgopapadakou and Tkacz, 1995; Munro, 2013). Additionally, the cell wall plays a crucial role in virulence and pathogenicity (Bulawa et al., 1995; Lenardon et al., 2010). The major cell wall components in oomycetes are cellulose, β-(1 → 3)- and β-(1 → 6)-glucans, but some species also produce small amounts of chitin (Bulone et al., 1992; Mélida et al., 2013). In *Saprolegnia monoica*’s hyphal cells, chitin occurs in the form of granular structures whereas regenerating protoplasts were shown to form microfibrils of chitin, suggesting that the environment in which chitin is deposited, i.e., the presence or absence of other cell wall components, influences its morphology and, most likely also, its physical properties (Bulone et al., 1992; Gay et al., 1992). The presence of chitin in oomycetes was used to group their cell walls in three main types based on the abundance of *N*-acetylglucosamine (GlcNAc), the monomer of chitin. Members of the oomycete order Peronosporales, which includes the important plant pathogen *Phytophthora infestans*, are devoid of GlcNAc and their cell walls belong to type I (Mélida et al., 2013). In contrast, species of the order Saprolegniales contain different levels of GlcNAc, i.e., up to 5% for type II or higher amounts for type III (e.g., ≈10% in *Aphanomyces euteiches*). The cell wall of *S. parasitica* belongs to type II, with approximately 2% chitin (Mélida et al., 2013). Despite the relatively small quantity of chitin in the Saprolegniales, previous research in our group on *S. monoica* suggests that the polymer is essential for cell wall integrity (Guerriero et al., 2010). Indeed, the data showed that the growth and morphology of *S. monoica* is strongly affected by the uridine-based nucleoside-peptide antibiotic nikkomycin Z (NZ), which is a specific inhibitor of yeast and fungal chitin synthases (CHS) (Gow and Selitrennikoff, 1984; Cabib, 1991; Gaughran et al., 1994; Kim et al., 2002). The bursting of *S. monoica*’s hyphal tips in the presence of this inhibitor suggests that chitin critically contributes to mechanical strength and resistance to intracellular turgor pressure at the apex of the cells, and plays an important role in mycelial tip growth in oomycetes (Guerriero et al., 2010). It was proposed that CHS proteins are located and most active at the tip of the hyphae and that chitin is transiently synthesized at the apex prior to the deposition of cellulose, the dominating polysaccharide in the subapical and mature parts of the cells (Guerriero et al., 2010).

Regardless of the content of chitin in the cell wall of different oomycetes, genes that code for predicted CHS have been found in all oomycete genomes analyzed to date (Klinter et al., 2019), suggesting that the putative *Chs* genes of oomycete species whose cell walls are devoid of chitin are either not functional or not involved in chitin biosynthesis. Two *Chs* genes were identified in *S. monoica*, with *SmChs2* being the most highly expressed in the mycelium (Guerriero et al., 2010). The catalytic activity of the protein *Sm*CHS2 and its inhibition by NZ were confirmed *in vitro*, providing the first evidence of an oomycete CHS that catalyzes the formation of chitin, and suggesting CHS as potential targets for anti-oomycete compounds (Guerriero et al., 2010). The *A. euteiches* genome also contains two *Chs* genes, but cell wall characterization of this species indicates that these genes are involved in the synthesis of either chitin with a low degree of polymerization (Mélida et al., 2013) or oligosaccharides that might be linked to other components of the cell wall to form heteroglycans (Badreddine et al., 2008; Nars et al., 2013). *P. infestans* contains one putative *Chs* gene (Haas et al., 2009), but analysis of its cell wall has been unable to detect any GlcNAc (Mélida et al., 2013). Nonetheless, the activity of the corresponding *Chs* product seems to be required for vegetative growth as the presence of NZ in the culture medium results in strong growth inhibition (Hinkel and Ospina-Giraldo, 2017) and tip bursting (Klinter et al., 2019). This was confirmed in recent work in other *Phytophthora* species where CHS proteins have been shown to be involved in vegetative growth, asexual reproduction, and pathogenesis (Cheng et al., 2019). Although the function of the *P. infestans Chs* gene remains elusive, its high expression during plant infection suggests that it has a specific role in pathogenesis (Hinkel and Ospina-Giraldo, 2017).

Altogether, these observations highlight the need to characterize oomycete CHS in greater detail to fully understand their function and eventually exploit these enzymes as targets for disease control. This is however complicated by the difficulty to generate mutants in this class of organisms and by the fact that most cell wall biosynthetic enzymes are vital. To circumvent this problem, we hereby report the use of a combination of *in silico*, molecular and biochemical approaches to characterize the *S. parasitica Chs* genes, quantify their expression, analyze CHS activity in the pathogen, and investigate the impact of NZ on chitin formation and hyphal growth. These data lay the basis for the further exploitation of chitin biosynthesis in the control of saprolegniosis.

## Materials and Methods

### Maintenance and Growth of *S. parasitica*

*Saprolegnia parasitica* (strain CBS 223.65, Centraal Bureau voor Schimmelcultures, Netherlands) was maintained on potato dextrose agar (PDA). For all experiments, mycelial plugs from the edges of 4-day-old colonies were used to inoculate either PDA or liquid Machlis medium (Machlis, 1953) as previously described (Bulone et al., 1992). All cultures were grown at 24°C in the dark.

### Treatment With Nikkomycin Z and Microscopy

Six-well culture plates containing either PDA or liquid Machlis medium supplemented with NZ (Sigma–Aldrich) at 0, 50, 200, and 800 μM were inoculated with 0.5 cm^2^ *S. parasitica* agar plugs and incubated at 24°C for 3 days. Mycelial growth was quantified on the PDA plates by measuring the diameters of the colonies once a day. The morphology of the hyphae growing in the liquid media was observed directly on the plates using a Leica DM IL LED inverted optical microscope. For determination of the effect of NZ on gene expression, *S. parasitica* mycelium was pre-grown in Machlis medium for 24 h before the addition of NZ to a final concentration of 800 μM. Samples were collected directly following the addition of NZ (0 h), and after 2, 6, 12, 24, and 48 h of incubation. For all time points, samples of mycelium not exposed to NZ were also collected and used as controls. All harvested samples were frozen in liquid nitrogen prior to further analyses.

### Determination of Chitin Content in Hyphal Cell Walls

To determine the impact of NZ on chitin content in the cell wall, the mycelium was pre-grown in liquid Machlis medium for 24 h prior to the addition of 0, 50, 200, or 800 μM NZ. The growth was continued for another 24 h and the samples were collected for GlcNAc quantification, which reflects chitin abundance as other forms of GlcNAc in the cell wall are negligible. Four milligrams of freeze-dried mycelial cell walls was prepared as described in Bulone et al. (1992) and added to 3 ml of 6 M HCl. The samples were heated at 100°C for 4 h and, after cooling to room temperature, the hydrolysates were filtered and evaporated to dryness at 50°C. The dry samples were dissolved in 1 ml of distilled water and assayed for GlcNAc content as described by Chen and Johnson (1983). Experiments were repeated on three independent biological replicates and standard deviations were calculated from duplicate GlcNAc assays performed on each biological replicate.

### Preparation of Microsomal Fractions, Protein Extraction, and Chitin Synthase Assays

Microsomal fractions were prepared by differential centrifugation from mycelium grown in Machlis liquid medium as previously described (Guerriero et al., 2010) and protein concentration in the samples was determined using the Bradford assay (Bradford, 1976). For membrane protein extraction, the final protein concentration of the microsomal fraction was adjusted to 4 mg/mL with extraction buffer (Tris–HCl 10 mM, pH 7.4), followed by the addition of one of three detergents (all from Sigma–Aldrich), i.e., 3-[(3-cholamidopropyl)dimethylammonio]-1-propanesulfonate (CHAPS), dodecylmaltoside (DDM), or digitonin, at final concentrations of 0.5 or 1% (w/v). The proteins were solubilized for 30 min with gentle stirring at 4°C. Following detergent extraction, the samples were centrifuged for 1 h at 100,000 × *g* and the supernatant was collected and used for enzymatic activity measurements. The *in vitro* CHS assays were performed in a final volume of 200 μl using either 500 μg of proteins from the microsomal fraction or 50 μl of detergent extract containing 50–60 μg of protein (Bradford assay). Reaction mixtures contained the following compounds (final concentrations in the 200-μl assay reactions): 0.5 mM UDP-*N*-acetyl-D-glucosamine, 14 nM UDP-*N*-acetyl-D-glucosamine [glucosamine-6-^3^H(N); 1.369 TBq/mmol; Perkin Elmer], 20 mM GlcNAc, 10 mM MgCl_2_, 10 mM Tris–HCl [tris(hydroxymethyl)aminomethane-*N*, *N*′-bis(2-ethanesulfonic acid)/2-amino-2-(hydroxymethyl)-1,3- propanediol hydrochloride] (pH 7.4), and trypsin as specified in the different assays presented in the section “Results.” After 1 h incubation at room temperature, the reactions were terminated by the addition of 400 μl of 95% ethanol. Following an overnight precipitation at −20°C, the reaction product was recovered on glass-fiber filters (Whatman GF/C) and washed with water and 95% ethanol under vacuum. The radioactivity incorporated into insoluble chitin was measured in a scintillation counter (Packard 1500 Tri-Carb) using 4 ml Ultima Gold scintillation cocktail (Bulone et al., 1992; Guerriero et al., 2010). The data were expressed as nmol of GlcNAc incorporated into chitin per mg of protein and standard deviations were calculated from three technical replicates.

### Enzymatic Characterization of the *in vitro* Product

The insoluble products synthesized *in vitro* in three separate reactions were pelleted by centrifugation at 10,000 × *g* for 10 min, washed with 1 ml of 75% ethanol, and resuspended in 100 μl of 50 mM potassium phosphate buffer (pH 6.0) containing 50 μg/ml chitinase from *Trichoderma viride* (≥600 units/g, Sigma–Aldrich). After a first incubation at room temperature for 24 h under continuous stirring, a fresh chitinase solution was added and a second 24-h incubation was performed. The hydrolysis was terminated by the addition of 400 μl of 95% ethanol. Samples exposed to phosphate buffer devoid of chitinase, but otherwise receiving identical treatment, served as controls. The non-hydrolyzed polysaccharides present in all samples were then precipitated overnight at −20°C, recovered on glass-fiber filters and quantified by scintillation counting as indicated above (Bulone et al., 1992; Guerriero et al., 2010).

### Sequence Determination and Bioinformatic Analysis of Chitin Synthases

The *Chs* gene and predicted protein sequences were retrieved from the *S. parasitica* draft genome sequence (Jiang et al., 2013) by BLAST algorithms using *S. monoica Chs1* and *Chs2* as query sequences. The sequences were downloaded from the Broad Institute website and are now available in NCBI^1^ (for accession numbers see Supplementary Table S1). For determination of the nucleotide sequences of *Chs*3-6, RNA was extracted from mycelium using the RNeasy plant mini kit (Qiagen).

cDNA was generated using the Maxima H Minus First Strand cDNA Synthesis kit with dsDNase (ThermoFisher Scientific). Genomic DNA (gDNA) was extracted from 3-day-old mycelium using the CTAB DNA extraction method (Murray and Thompson, 1980). Specific areas from cDNA and gDNA were amplified using the Phusion High-Fidelity DNA polymerase (ThermoFisher Scientific) according to the manufacturer’s recommendations and the primer pairs listed in Supplementary Table S2. The thermal cycling conditions were as follows: initial denaturation at 98°C for 30 s; 35 cycles at 98°C for 5 s; annealing temperature (60 or 65°C) for 10 s; 72°C for 10 s; final extension step of 72°C for 10 min. PCR products obtained were purified, sequenced by Eurofins Genomics (Germany), and compared with the sequences downloaded from the Broad Institute database by alignment, using the Multalin software^2^. The new nucleotide sequences were translated into amino acid sequences using the ExPaSy software^3^. Putative protein domains were predicted with the NCBI CDD search program^4^ (Marchler-Bauer et al., 2010). The CCTOP v1.00^5^ (Dobson et al., 2015) algorithm was used to predict transmembrane domains. For sequence comparison with other oomycete CHS proteins, sequences were retrieved from selected genomes by sequence similarity searches using blastP against *S. monoica* CHS1 and CHS2. The sequences were downloaded from the NCBI (see text footnote 1) and JGI genome^6^ portals. The following oomycete CHS sequences were selected: CHS from *S. monoica* (*Sm*CHS1, ADE62520; *Sm*CHS2, ADE62521), *A. euteiches* (*Ae*CHS1, ACA96933; *Ae*CHS2, ACA49151), and *P. infestans* (*Pi*CHS, XP_002908631). Shared identities between full-length CHS proteins were calculated using EMBOSS Needle^7^ (Rice et al., 2000). Putative glycosylation and phosphorylation sites were identified using NetNGlyc 1.0 and NetPhos 2.0^8^, respectively. Alignment of the six *Sp*CHS amino acid sequences with *Sm*CHS1 and 2, the *Saccharomyces cerevisiae Sc*CHS2 and *Neurospora crassa Nc*CHS2 was completed using ClustalOmega^9^ (Sievers et al., 2011).

### Southern Blot Analysis

For Southern blot analysis, mycelium grown in Machlis medium for 3 days was frozen in liquid nitrogen and finely ground with a mortar and pestle. Ten micrograms of gDNA isolated as described above was incubated with 0.5 μg RNase A and digested with FastDigest restriction enzymes following the manufacturer’s instructions (ThermoFisher Scientific). The resulting fragments were separated by electrophoresis on 0.8% agarose gels and transferred onto a nylon membrane (Sambrook et al., 1989). The *SpChs* probe was generated by PCR amplification (Phusion High-Fidelity DNA polymerase, ThermoFisher Scientific) from a 212 nucleotide sequence of *SpChs6* (1822-2033 nt) which shared a minimum 79% identity with the remaining *SpChs* genes. The following primers were used: Fwd, 5′-GGCAACATGTACCTCGCCGAAGATCGC-3′; Rev, 5′-GTGTAAACGCGGCCCCAGTTCAGAATC-3′. The PCR products were cloned in pBluescript II SK vector (ThermoFisher Scientific) and transformed in *Escherichia coli* TOP10 (Invitrogen) following the manufacturers’ instructions. Plasmids were extracted (GeneJET Plasmid Miniprep Kit, ThermoFisher Scientific) and the cloned sequence verified by DNA sequencing (Eurofins Genomics, Germany). The plasmids were then used as template for the generation of the PCR labeled probe using the PCR DIG Probe Synthesis kit (Roche). To this end, 10 pg of plasmid and primers (1 μM) were included in the PCR reactions, which were conducted using the following conditions: initial denaturation at 95°C for 2 min, followed by 30 cycles of 95°C for 30 s, 64°C for 30 s, and 72°C for 40 s, and a final extension step of 72°C for 7 min. Membrane prehybridization and hybridization were carried out in 5 ml of DIG Easy Hyb buffer (Roche) at 42°C. Membranes were prehybridized during 30 min, followed by the addition of the PCR-labeled probe at 2 μl/ml final concentration. After 16 h hybridization, blots were first washed twice with 2× SSC buffer (300 mM NaCl, 30 mM Na-citrate pH 7) containing 0.1% sodium dodecyl sulfate (SDS), for 5 min at room temperature, and then washed twice with 0.5× SSC buffer containing 0.1% SDS for 15 min at 65°C. Immunodetection was performed using the DIG Luminescent Detection Kit (Roche) and the signal developed after exposure of the blots to X-ray films.

### qPCR Analysis

Expression of the six *Chs* genes was analyzed by qPCR. Frozen mycelium was ground to a fine powder with a mortar and pestle and 100 mg material was used for total RNA extraction using the RNeasy Plant Mini Kit (Qiagen). Trace amounts of contaminant DNA was removed using the Turbo DNA-free kit (Life Technologies) according to the manufacturer’s instructions. cDNA was synthesized from 1 μg of DNA-free RNA in a reverse transcription reaction (iScriptcDNA synthesis kit, BioRad). The CFX96 Real-Time PCR detection system (BioRad) was used for qPCR experiments. The expression stability of six potential reference genes in our set of samples was analyzed by the statistical algorithm geNorm (Vandesompele et al., 2002). Three genes were selected as the most stable [elongation factor 1-α (SPRG_10439), γ-tubulin (SPRG_10729), and ubiquitin-conjugating enzyme E2 (SPRG_03371)] and used as reference genes. Specific primers for each of the six *Chs* genes and reference genes are listed in Supplementary Table S2. For all genes examined, two biological replicates were included in the qPCR analysis and all amplification reactions were performed in triplicate in a total volume of 10 μl containing 10 ng cDNA, 0.75 μM of each primer, and 1× iQ SYBR green Supermix (BioRad). Controls lacking reverse transcriptase or template were included. The thermal cycling conditions were as follows: initial denaturation at 95°C for 3 min, followed by 40 cycles of 95°C for 10 s, 58°C for 10 s, and 72°C for 10 s. Fluorescence was measured at the end of the extension step. An independent samples *t*-test was used to determine two-tailed significance of the expression level for a target gene between the control samples and samples treated with NZ.

## Results

### Growth Inhibition of *S. parasitica* by Nikkomycin Z

**FIGURE 1:**
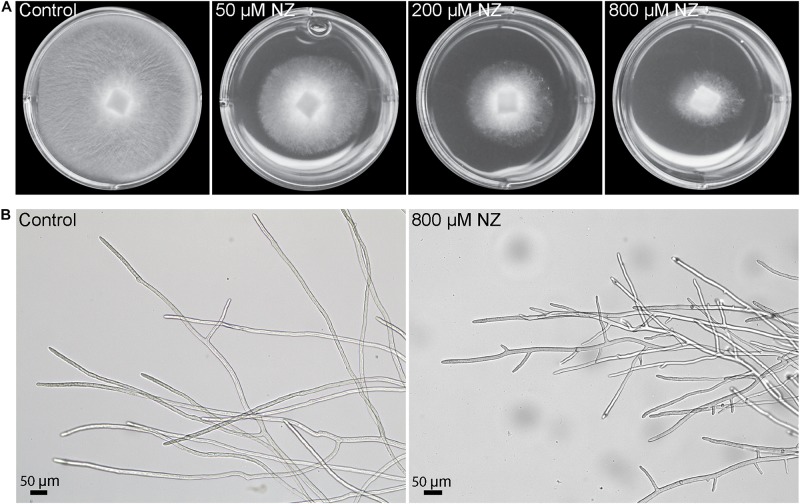
Sensitivity of *S. parasitica* to nikkomycin Z (NZ). **(A)** Mycelial growth following 2 days culture on PDA medium supplemented with 0, 50, 200, and 800 μM NZ. **(B)** Optical micrographs of hyphal cells following 2 days culture in Machlis medium devoid of NZ **(left)** or supplemented with 800 μM NZ **(right)**.

Nikkomycin Z is a specific inhibitor of CHS and it was shown to inhibit the growth of the oomycete *S. monoica* (Guerriero et al., 2010). To test whether this antibiotic has a similar effect in the growth of the devastating fish pathogen *S. parasitica*, the mycelium was grown in the presence of three different concentrations of NZ. After 48 h incubation on solid medium, the growth of *S. parasitica* was reduced by ∼40% at the lowest NZ concentration of 50 μM and a stronger inhibition of around 65% was observed at 800 μM NZ (Figure 1A). In addition, in the presence of NZ, the mycelium mat appeared thinner than in the control wells (Figure 1A) and it consisted of highly branched hyphae which are typically not observed in the control devoid of NZ (Figure 1B).

### Effect of Nikkomycin Z on Chitin Synthase Activity

**FIGURE 2:**
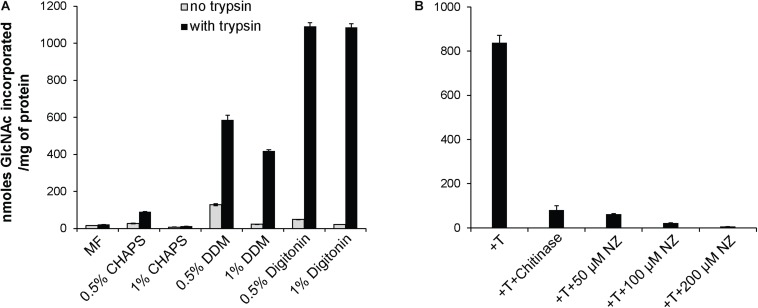
*In vitro* radiometric assays of chitin synthase activity. **(A)** CHS activity from *S. parasitica* microsomal fractions (MF) and membrane proteins extracted with 0.5 or 1% CHAPS, DDM, or digitonin. **(B)** Detection of insoluble product in 0.5% digitonin extracts in the presence of trypsin (T), and different concentrations of NZ and chitinase. The data from a representative experiment are presented and error bars correspond to the standard deviations calculated from three technical replicates of the experiment shown.

The observed mycelial growth inhibition by NZ prompted us to confirm the presence of CHS activity in the mycelium of *S. parasitica* and to investigate how NZ affects the enzymatic activity. To this aim we performed *in vitro* enzymatic assays and quantified the incorporation of radioactive GlcNAc from radiolabeled UDP-*N*-acetylglucosamine into insoluble chitin. Radioactive insoluble product was easily detected, demonstrating that *S. parasitica*’s mycelial enzymes are active *in vitro*, especially in the presence of trypsin (Figure 2A). In an effort to enrich native CHS activity, we tested the ability of three different detergents, CHAPS, DDM, and digitonin, to extract the enzymes from the membranes while preserving the activity. When DDM or digitonin was used for protein extraction, the CHS activity in the detergent-soluble fractions was highly enhanced in the presence of trypsin (Figure 2A). Specifically, proteolytic activation in 0.5 and 1% digitonin extracts increased the enzymatic activity around 20 and 50 times, respectively, compared to the same assays performed in the absence of trypsin. A similar but smaller effect was observed using DDM. The least effective detergent tested was CHAPS (Figure 2A). Based on these results, we proceeded with the membrane protein extractions in 0.5% digitonin for further experiments. The radioactive product synthesized *in vitro* was sensitive to chitinase, which confirmed the formation of polymeric chitin in the assay conditions used (Figure 2B). Inhibition by NZ was concentration dependent as the amount of radioactive product synthesized decreased with increasing concentrations of NZ, reaching an almost complete inhibition of chitin formation at a NZ concentration of 200 μM (Figure 2B). These results further confirm the specificity of the inhibitory effect of NZ on the CHS enzymes from *S. parasitica* (Figure 2B).

### *In silico* Characterization of the *SpChs* Genes

**FIGURE 3:**
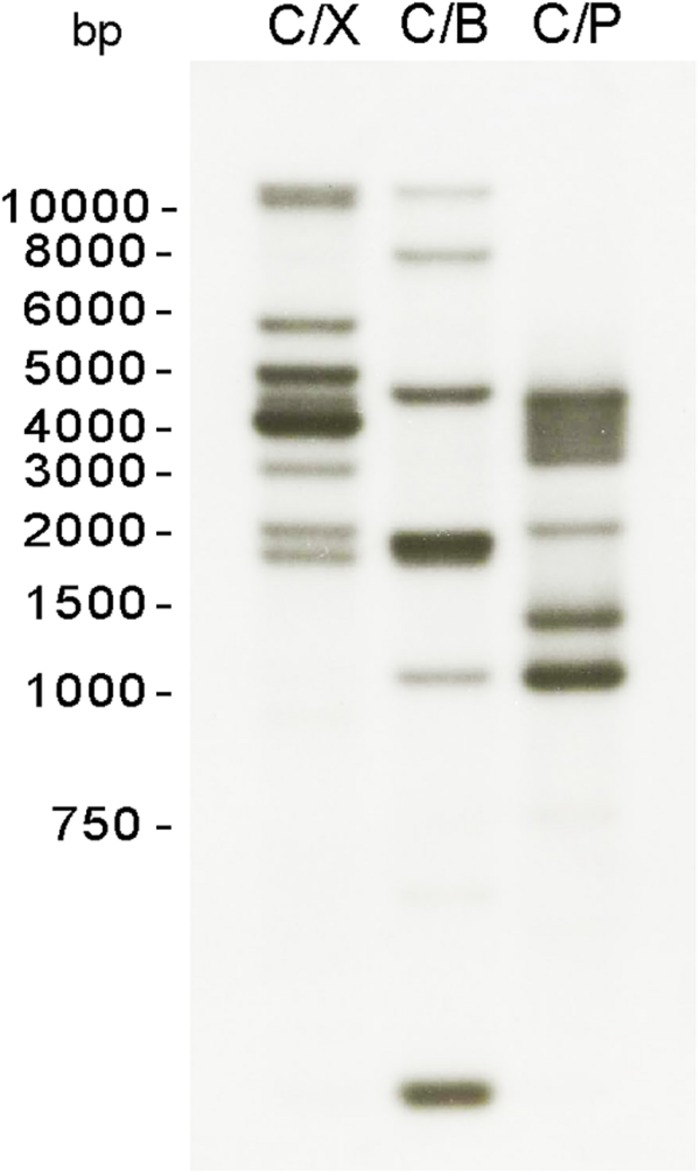
Southern blot analysis of *S. parasitica Chs* genes. Genomic DNA was digested with a combination of two of the following enzymes: *Cla*I (C), *Xba*I (X), *Pst*I (P), *Bgl*I (B), and transferred to nylon membranes. A probe corresponding to a conserved region of all *SpChs* genes was used for the hybridization of the membrane.

Given the CHS activity observed in *S. parasitica* mycelium, we searched the draft genome sequence available in the Broad Institute database for putative *Chs* genes. Six genes were found to encode proteins with CHS domains (Jiang et al., 2013) and were designated *Chs*1 to *Chs*6 (Supplementary Table S1). The predicted amino acid sequences were compared to that of non-redundant protein sequences (nr) in the NCBI database using the BlastP algorithm. Notable differences were found in CHS3, CHS4, CHS5, and CHS6 relative to other oomycete CHS proteins. More specifically, CHS4 and CHS6 had longer N-terminal ends, while CHS3 and CHS5 contained deletions. To further our analysis, we amplified and sequenced the genomic and cDNA regions corresponding to these genes, where potential errors in the sequence determination could have occurred. *Chs*4 and *Chs*6 sequences contained an extra nucleotide compared with the sequences retrieved from the database, while *Chs*3 and *Chs*5 presented longer sequences that had been previously predicted as introns. The new coding sequences were determined and submitted to sequence comparison using BlastX. Relative to the previous protein sequences found in the database, our updated sequences showed increased similarity with the CHS proteins of other oomycetes. The new predicted amino acid sequences of CHS3, CHS4, CHS5, and CHS6 were included in our analysis and can be found in Supplementary Table S1. To confirm the number of *Chs* genes in *S. parasitica*, we performed a Southern blot analysis (Figure 3). A conserved region in all six *Chs* genes was selected to design a *SpChs* probe (section “Materials and Methods”). In agreement with the number of *Chs* genes identified in the draft genome, six bands were detected following digestion of the *S. parasitica* gDNA with *Cla*I/*Bgl*I (Figure 3). However, eight bands were detected following *Cla*I/*Xba*I digestion. As no restriction site was identified in the probe binding regions of the six *SpChs* genes, our results suggest the presence of more than six *Chs*-related sequences in the genome of *S. parasitica.* However, due to the heterozygous nature of some *Chs* genes, with 65–83 SNPs detected (Jiang et al., 2013), a likely possibility is that *Chs* allelic variations introduced additional *Xba*I restriction sites in *SpChs* loci, thereby leading to the detection of more than one hybridization band per gene. The number of bands was unclear following *Cla*I/*Pst*I digestion (Figure 3).

**FIGURE 4:**
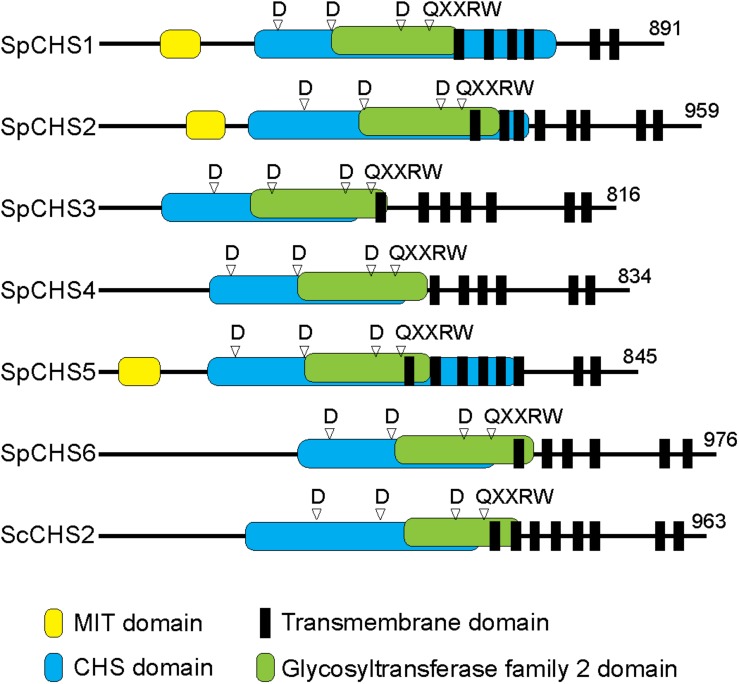
Predicted domain organization of the six *S. parasitica* CHS proteins compared with CHS2 from *S. cerevisiae* (Sc). All proteins contain CHS and GT2 domains, including the conserved amino acid residues D, D, D and QXXRW motif. *Sp*CHS1, *Sp*CHS2, and *Sp*CHS5 carry a microtubule interacting and trafficking domain (MIT) at their N-terminal ends. Predicted multiple transmembrane domains are located toward the C-terminal ends of the proteins.

Chitin synthases and transmembrane domains were predicted in the encoded amino acid sequences and the full-length sequences were compared with that of the well-characterized CHS2 from *S. cerevisiae* (Figure 4). All CHS analyzed share a similar domain organization. Indeed, all of the CHS contain CHS domains (pfam01644, pfam03142, pfam08407) and glycosyltransferase family 2 (GT2) domains (pfam13632) that carry the conserved yeast and fungal CHS motifs (motifs a–h) (Supplementary Figure S1; Ruiz-Herrera et al., 2002; Choquer et al., 2004; Badreddine et al., 2008). Moreover, the putative catalytic domain includes three conserved spaced aspartate residues (D, D, D) and the pentapeptide QXXRW present in most processive glycosyltransferases (Saxena et al., 1995; Campbell et al., 1997). Further, all of the CHS proteins have six to eight predicted transmembrane domains located at their C-terminal ends. In contrast to other *S. parasitica* CHS, *Sp*CHS1, *Sp*CHS2, and *Sp*CHS5 contain a microtubule interacting and trafficking (MIT) domain (pfam04212 and cd02656) (Ciccarelli et al., 2003). The examined *SpChs* genes encode proteins with molecular weights ranging from 91 to 108 kDa (Supplementary Table S3) and all enzymes have potential phosphorylation sites, while *N*-glycosylation was predicted for only CHS1, CHS2, and CHS3 (Supplementary Table S3 and Supplementary Figure S1).

**FIGURE 5:**
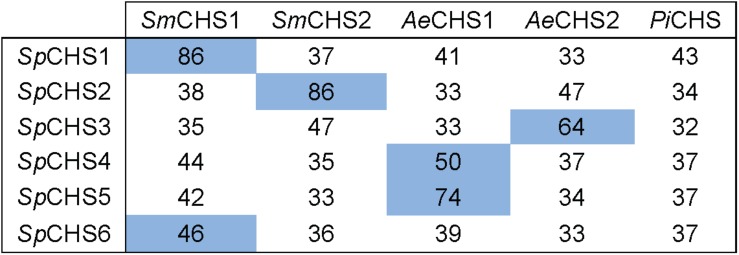
Amino acid identity matrix between *Sp*CHS and CHS from selected oomycete species. Sm, *Saprolegnia monoica*; Ae, *Aphanomyces euteiches*; Pi, *Phytophthora infestans*. The percentage of amino acid identity was calculated for the full-length sequences. The highest percentage of identity for each *Sp*CHS is highlight in blue.

In an effort to compare sequence identify of orthologs of individual *S. parasitica* CHS from other oomycete species, CHS previously identified in the Saprolegniales (*S. monoica* and *A. euteiches*) (Badreddine et al., 2008; Guerriero et al., 2010) and Peronosporales (*P. infestans*) (Hinkel and Ospina-Giraldo, 2017) orders were selected. The percentages of identity of *Sp*CHS full-length amino acid sequences to the CHS of *S. monoica*, *A. euteiches*, and *P. infestans*, are presented in Figure 5. Two of the *Sp*CHS share 86% identity with CHS1 and CHS2 of *S. monoica*, and were designated as *Sp*CHS1 and *Sp*CHS2, respectively, to match the corresponding *S. monoica* proteins. The remaining four *Sp*CHS did not share particularly high identity with any of the *S. monoica* CHS and were consequently designated CHS3, CHS4, CHS5, and CHS6. *Sp*CHS4 and *Sp*CHS5 were most closely related to *Ae*CHS1 with 50 and 74% identity, respectively. We found the closest sequence of *Sp*CHS6 to be *Sm*CHS1 (46% identity) while *Sp*CHS3 is most closely related to *Ae*CHS2 (64% identity).

### Effect of NZ on Expression of *SpChs* Genes and Chitin Content

**FIGURE 6:**
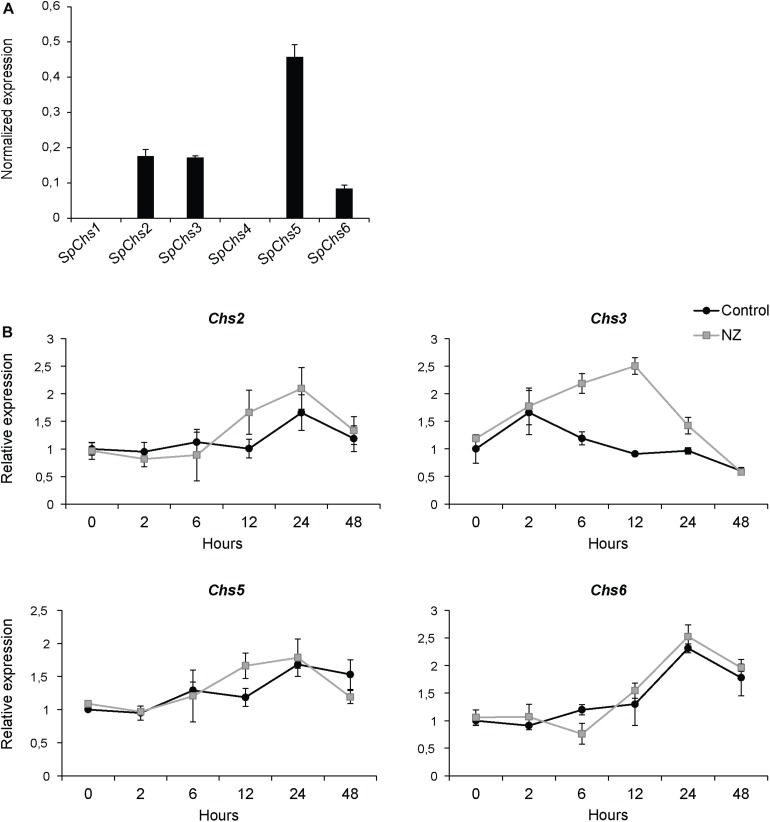
Expression analysis of *SpChs* genes in mycelium. Gene expression was measured after 48 h of growth **(A)** and the effect of NZ on expression levels was measured at 0, 2, 6, 12, 24, and 48 h after addition of 800 μM inhibitor in the medium (final concentration) or water (control) **(B)**. Error bars represent standard deviations calculated from triplicate qPCR reactions performed on each of two biological replicates.

**FIGURE 7:**
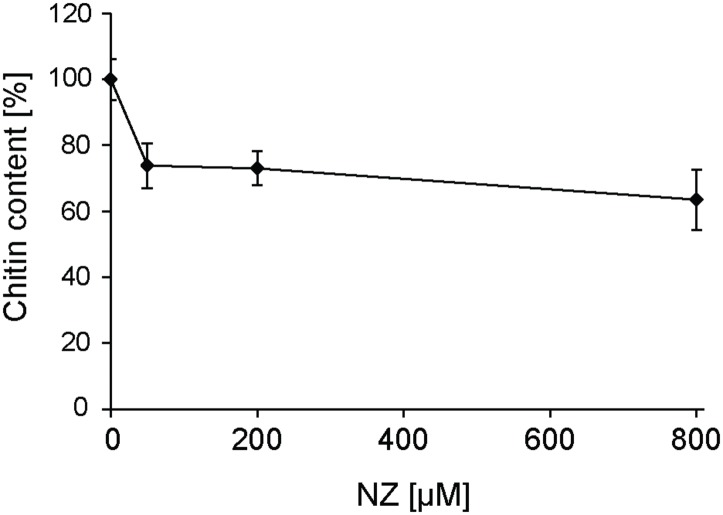
Effect of different concentrations of NZ on the chitin content in *S. parasitica*’s mycelial cell walls measured 24 h after addition of the inhibitor in the culture medium. One hundred percent correspond to the chitin content measured in the control (0 μM NZ). Error bars represent standard deviations calculated from technical GlcNAc assays performed in duplicate on each of three biological replicates.

The expression of the six *SpChs* genes in the mycelium was investigated by qPCR analysis. Our results demonstrate that *Chs 2*, *3*, *5*, and *6* are expressed in the mycelium, where *Chs5* has the highest expression. On the contrary, *Chs1* and *4* did not exhibit detectable levels of expression (Figure 6A). The effect of NZ on expression levels was investigated at different time points over 48 h (Figure 6B). NZ was added following 24 h of mycelial pre-growth. The expression levels varied across measured time points with *Chs2*, *Chs5*, and *Chs6* showing similar expression profiles, with higher expression after 24 and 48 h, whereas the highest level of expression of *Chs*3 was observed after 2 h contact with NZ. Interestingly, *Chs3* was the only gene significantly affected by NZ (*P* < 0.05). Relative to the corresponding controls in the absence of NZ, the highest increase in the expression level of *Chs3* was detected 6–12 h after the addition of NZ, reaching a threefold up-regulation of *Chs3* mRNA 12 h after addition of the inhibitor. The difference in expression level observed between the control and NZ-treated samples decreased within 24 h and no difference was measurable after 48 h treatment. As *Chs3* was the only gene whose expression increased in the presence of NZ, it can be suggested that it is involved in a compensatory mechanism to cancel the effects of CHS inhibition. However, the higher expression of *Chs3* together with the expression of the other *Chs* genes does not seem to be sufficient to completely overcome chitin deficiency in the cell wall and the resulting effects on mycelial growth (Figure 1) as chitin content was reduced by nearly 25 and 40% after 24 h growth in the presence of 50 and 800 μM NZ, respectively (Figure 7).

## Discussion

*Saprolegnia parasitica* is a devastating fish pathogen responsible for significant economic losses in the aquaculture industry globally. Despite being a minor component of oomycete cell walls, chitin plays an important role in maintaining cell wall integrity (Guerriero et al., 2010). It follows that the enzymes responsible for chitin biosynthesis, CHS, represent potential targets for disease control. Our *in silico* search of the *S. parasitica* genome draft sequence resulted in the prediction of six *Chs* genes. Despite our Southern blot analysis suggested the presence of up to eight *Chs* genes (Figure 3), the detection of more than six bands is most likely due to allelic variations at the heterozygous loci. The presence of six *Chs* genes in *S. parasitica* is also supported by the identification of six *Chs* genes in other species of the order Saprolegniales (Klinter et al., 2019). Interestingly, the recent phylogenetic analysis of 93 oomycete *Chs* products divided Saprolegniales CHS into six different clades. According to this study, each of the CHS identified in *S. parasitica* belongs to a separate clade (Klinter et al., 2019), indicating they are more similar to their orthologs than to their paralogs.

The predicted amino acid sequences of all six *Sp*CHS share a similar domain organization, including the presence of CHS domains and multiple transmembrane domains toward the C-terminal ends of the proteins (Figure 4). Interestingly, the structural differences between the six *Sp*CHS proteins in terms of the presence or absence of an N-terminal MIT domain, the position of the MIT domain, or the length of the full-length proteins is also conserved in the corresponding orthologs from other species (Klinter et al., 2019). The CHS domains consistently contain all putative amino acids expected to be required for enzyme activity (Supplementary Figure S1). Moreover, all *Sp*CHS share a common protein structure with the *S. cerevisiae* CHS2, for which the function has been confirmed (Sburlati and Cabib, 1986; Silverman et al., 1988; Shaw et al., 1991). These results indicate that all six *SpChs* genes identified here are potentially functional. Of the six *S. parasitica* CHS, only CHS1, CHS2, and CHS5 were predicted to contain a MIT domain (Figure 4). However, the presence of MIT domains in other *Sp*CHS cannot be ruled out as suggested by the application of other bioinformatic analyses which predicted the presence of MIT domain in all *Sp*CHS, with the exception of *Sp*CHS3 (Klinter et al., 2019). MIT domains are known to be involved in the intracellular trafficking of proteins in many species (Ciccarelli et al., 2003). Interestingly, the oomycete CHS proteins are currently the only known processive carbohydrate synthases where MIT domains have been found. The functional characterization of the MIT domain from *S. monoica* CHS proteins suggested this domain is involved in the intracellular transport and/or regulation of CHS enzymes (Brown et al., 2016). In fungal CHS a similar role to MIT domain is played by the myosin motor domain (MMD), which is involved in apical exocytosis. It was demonstrated that in *Ustilago maydis* two different CHS co-travel to the plasma membrane in the same secretory vesicle, where only one CHS contains an MMD (Schuster et al., 2016). By analogy with fungal CHS and the associated MMD domains, it can be speculated that the oomycete CHS that lack an MIT domain are co-delivered to the plasma membrane together with one or several paralog that carry an MIT domain (Klinter et al., 2019). However, further experimental work is needed to confirm the presence of functional MIT domains in *Sp*CHS and their function.

The different *Chs* of *S. parasitica* likely have diverse biological functions, perhaps comparable to those previously described in the filamentous fungus *Magnaporthe oryzae* (Kong et al., 2012) or the yeast *S. cerevisiae* (Cabib et al., 1989; Shaw et al., 1991). The *M. oryzae* genome contains seven *Chs* genes known to play distinct roles in appressoria formation, pathogenesis, hyphal growth, and conidiogenesis (Kong et al., 2012). Likewise, *S. cerevisiae* contains three *Chs* genes that have diverse functions in cell wall repair, septum formation, and the formation of a chitin ring during budding (Cabib et al., 1989; Shaw et al., 1991). We found that only *Chs*2, *Chs3*, *Chs5*, and *Chs6* are expressed in the mycelium of *S. parasitica*, indicating an involvement of these genes in growth or other processes during the vegetative phase, while the remaining two *Chs* genes (*Chs1* and *Chs4*) may have a function during other life cycle phases or processes. This is in agreement with RNA-sequencing data (Jiang et al., 2013) that indicate *Chs1* and *Chs4* are expressed in cysts and germinating cysts. Given the similarity of *Sp*CHS1 and *Sp*CHS2 to *Sm*CHS1 and *Sm*CHS2, respectively, it is probable that they share common functions. Thus, *Sp*CHS2, like *Sm*CHS2, could potentially produce crystalline chitin in the mycelium and be involved in tip growth. Moreover, the highest expression of *Chs5* in the mycelium suggests this gene is responsible for the synthesis of most of the mycelial chitin.

In the present work, chitin biosynthesis in *S. parasitica* mycelium was investigated. Our *in vitro* activity assays demonstrated that membrane proteins from the mycelium do indeed produce ethanol-insoluble chitin. The identity of the reaction product was confirmed by hydrolysis with a chitinase (Figure 2B). The activity of CHS was detected in both the microsomal fraction and the detergent extracts (Figure 2A). The highest activity was observed in detergent extracts prepared using 0.5% DDM or 0.5% of digitonin, which are effective detergents for solubilizing membrane proteins with a good activity recovery (Aloni et al., 1983; Gay et al., 1993; Machida and Saito, 1993). Our data also suggest that trypsin activation is more effective on detergent-extracted proteins than on membrane-bound proteins. One possible explanation for the higher activation of detergent-extracted enzymes is improved access of trypsin to the protease cleavage site, which may be masked on the proteins in the microsomal fraction. The same type of proteolytic activation was observed previously in different CHS (Duran and Cabib, 1978; Sburlati and Cabib, 1986; Gay et al., 1989; Bulone et al., 1992). Interestingly, in *S. cerevisiae* the stimulatory effect of trypsin was detected only for *Sc*CHS1 (Duran and Cabib, 1978; Orlean, 1987) and *Sc*CHS2 (Sburlati and Cabib, 1986; Uchida et al., 1996; Martínez-Rucobo et al., 2009), but not for *Sc*CHS3, whose activity was found to be partially reduced by prior treatment with the protease (Orlean, 1987). The precise effect of trypsin on the individual *S. parasitica* CHS present in the mycelium cannot be elucidated as our *in vitro* assays were performed using total protein extracts. However, the observed 20-fold increased activity in the presence of trypsin (Figure 2A) implies a zymogenic character for at least some of the CHS and their activation by partial proteolysis. Trypsin was shown to exert an indirect effect on CHS activity in *S. cerevisiae* where it activates *in vitro* an unknown endogenous soluble protease, which subsequently stimulates the activity of *Sc*CHS2 (Martínez-Rucobo et al., 2009). Thus, proteolysis might be one of the mechanisms involved in the *in vivo* regulation of *Sp*CHS, where trypsin may be substituted by another endogenous protease. In addition to proteolytic activation, our *in silico* analysis revealed the presence of potential phosphorylation sites in all *Sp*CHS (Supplementary Table S3), suggesting phosphorylation may be another regulatory mechanism of these enzymatic activities.

Motivated by the inhibitory effect of NZ shown in previous studies on CHS, we also investigated the effect of NZ on *in vitro Sp*CHS activity. The results revealed a 14-fold reduction of CHS activity in digitonin extracts in the presence of 50 μM NZ, and almost complete inhibition in the presence of 200 μM NZ (Figure 2B). The inhibitory effect of NZ on CHS was previously described on yeast, fungal, and oomycete CHS (Gow and Selitrennikoff, 1984; Cabib, 1991; Gaughran et al., 1994; Kim et al., 2002; Guerriero et al., 2010). However, the different *S. cerevisiae* CHS isoforms varied in susceptibility to the antibiotic. *Sc*CHS1 and *Sc*CHS3 were inhibited by NZ, while *Sc*CHS2 was barely affected (Cabib, 1991; Gaughran et al., 1994). In contrast, all three corresponding CHS isozymes from *Candida albicans* were sensitive to NZ treatment (Kim et al., 2002). Our results indicate that all *Sp*CHS active *in vitro* are affected by NZ as the activity recovered from the mycelium was almost entirely abolished by the antibiotic.

The inhibitory effect of NZ on CHS *in vitro* was correlated with a physiological effect and a decreased abundance of chitin in the cell wall (Figure 7). A reduction in mycelial growth was apparent at a modest concentration of 50 μM NZ (Figure 1A), which makes *S. parasitica* more susceptible to the drug than the previously investigated *S. monoica* (Guerriero et al., 2010). The increased susceptibility of *S. parasitica* to NZ further substantiates the importance of chitin biosynthesis in cell wall integrity in this pathogen. At 800 μM NZ, the highest tested concentration, the growth of *S. parasitica* was greatly reduced, but not completely abolished (Figure 1A). The inhibitory effect of NZ on growth might possibly be overcome by up-regulation of *Chs* gene expression to produce sufficient active protein and overcome the biochemical inhibition. Indeed, we observed that the expression of *Chs3* was particularly up-regulated, perhaps indicating an important role for this protein in mycelial growth (Figure 6) and probably a partially redundant function with other *Sp*CHS. This effect appears to be very specific as NZ did not affect the expression of any other genes related to the synthesis of cell wall components, like cellulose synthases (Rzeszutek et al., unpublished results). Following 2 days incubation with 800 μM NZ, we observed an enhanced branching of hyphal cells (Figure 1B). Similar morphological alterations have been observed in *Aspergillus fumigatus* (Mellado et al., 1996) and *Aspergillus nidulans* (Borgia et al., 1996) CHS mutants. Indeed, the disruption of *chsG* and *chsB* genes in *A. fumigatus* and *A. nidulans*, respectively, resulted in reduced radial growth and highly branched hyphae, indicating apical localization of those enzymes and an important role during hyphal growth (Borgia et al., 1996; Mellado et al., 1996). Thus, our results suggest that NZ also has an inhibitory effect on CHS activity *in vivo* and that *Chs3* is overexpressed in response to disrupted chitin biosynthesis. Under this condition, CHS3 may be responsible for the formation of new mycelial tips leading to the observed branching phenotype.

## Conclusion

Our work demonstrates the importance of chitin in the physiology of *S. parasitica*. The inhibition of CHS by NZ leads to an aberrant phenotype and reduced growth of the pathogen, through alteration of cell wall formation. This conclusion is substantiated by the strong inhibitory effect of CHS activity observed *in vitro*. Altogether, our results support the potential of using oomycete CHS as drug targets to tackle the pathogen. However, a detailed functional characterization of each of the described enzymes is required to design inhibitors with increased efficiency.

## Data Availability Statement

The datasets generated for this study can be found in the NCBI website (protein accession numbers are provided in Supplementary Table S1).

## Author Contributions

VB designed and supervised the research, which was performed by ER and SD-M, and revised the manuscript. All authors contributed to the data analysis and interpretation. ER wrote the first draft of the manuscript with input from all authors.

## Conflict of Interest

The authors declare that the research was conducted in the absence of any commercial or financial relationships that could be construed as a potential conflict of interest.

## Footnotes

1 http://www.ncbi.nlm.nih.gov/

2 http://multalin.toulouse.inra.fr/multalin/

3 http://web.expasy.org/translate/

4 http://www.ncbi.nlm.nih.gov/Structure/cdd/wrpsb.cgi

5 http://cctop.enzim.ttk.mta.hu/

6 https://genome.jgi.doe.gov/portal/

7 http://www.ebi.ac.uk/Tools/psa/emboss_needle/

8 https://services.healthtech.dtu.dk

9 http://www.ebi.ac.uk/Tools/msa/clustalo/
