# Supplementary material for: Identification and Characterization of the Chitin Synthase Genes From the Fish Pathogen Saprolegnia parasitica
Source: Front Microbiol. 2019 Dec 13;10:2873. doi: 10.3389/fmicb.2019.02873 (PMC6923183; doi:10.3389/fmicb.2019.02873)
Supplement: Supplementary file 1 [file Data_Sheet_1.pdf]

Supplementary material

Identification and characterization of the chitin synthase genes from the fish pathogen *Saprolegnia parasitica*

Elzbieta Rzeszutek[1], Sara M. Díaz-Moreno[1] and Vincent Bulone[1,2,*]

[1]KTH Royal Institute of Technology, School of Engineering Sciences in Chemistry, Biotechnology and Health, Department of Chemistry, Division of Glycoscience, AlbaNova University Centre, 106 91 Stockholm, Sweden
[2]ARC Centre of Excellence in Plant Cell Walls and School of Agriculture, Food and Wine, The University of Adelaide, Waite Campus, Urrbrae, SA 5064, Australia

*Correspondence:
Vincent Bulone, E-mail: bulone@kth.se

Table S1. Sequences of *S. parasitica* chitin synthases.

BROAD Institute accession number	NCBI protein accession number[1]	Name	Protein sequence
SPRG_09812	XP_012204852	*S. parasitica* CHS1	MPPKRPTEASGRRYAPPAGRPSNNAANAKPRAPRKGVSSRASNVPSAASSYEYDYEYNMM<br>PMMQAPPKSQPTFLSNIAPISAKEASMKGSNAMQLLLQGTSFTIDDAFRAIERAIQAENE<br>GRFREALKHFLDGGEMIVTAAEKEASQKVRNLLLHKGKEVLEWAEHLAEWIERYNTSTPP<br>VRIAKPMAVEVTYDRTMNSPDLDETEARMMFYTPVCSGPKAFTETGYRLQCIQSGRRPRL<br>MVVITMYNEDENELRSTLRKVCNNVLYLKQHSLPGYEGDDAWKQVLVVVVSDGRTKANKG<br>TLEWLANVGLYDEDVMNITSTGVKVQCHLFEHSLQMTKENSIRFPPLQLDSHLWYFDAFA<br>EQIMPDYTVLLDVGTMPTKSSFYKLLTALEINAQIGGVCGEIAVDKPLPNMCNWVIAAQH<br>FEYKISNILDKSLESCFGFISVLPGAFSAYRYKAIRGAPLQAYFKSLTTDMAELGPFAGN<br>MYLAEDRILCFELLARKDCNWTMHYVKDAIARTDVPTNLIDLVGQRRRWLNGSFFATLFA<br>IWNWGRVYTESNHSLTRKLALLVHALLGVSAANFYLALYFVIFQGFRDNRWNFIDTSEYP<br>QWVLDGLPTAFNVFYAVTVFTQVTIGLGNKPKHVKGTHYLISVLFGLLMLLASGVAIVIF<br>ITSSKDAMAIVLAVLILGTFFIGSALHCEVHHIVLTFVQYTALMPSFVNILMVYSFCNLH<br>DLSWGTKGIDTGHEAHKTEAVGQYKDIVARQKALEAKKAQDARNQDELKKRFDSFRSNLL<br>LVWVMSNMSMVIICVNTVGADSFLPFLYAFVAAFNGIRLLGCIGYLIYYARQFLLFNTLS<br>ATGVLHKRHEARKHKKAEDPDPIDMELGTFNEPATSEIGAPMMQAPYNRMR
SPRG_04151	XP_012198147	*S. parasitica* CHS2	MSDSNLDLAARLRALREGGAEPAPAPAPTPYMHSPPSRTRPTPLYTQESLEFGGTYTTGS<br>PVGAEADGVYTQVPVWKDSKEKTYGYLDDEPAPQAQTLLNKANDLVQRQASNKAFRRQHT<br>AAFRPLPNTVEELLDGSPTYEGAFRLVQLAVQMEQDGDPQAAINLYADAGATLVEVGRKE<br>VDPLLQKGIRQKAQELLQRAEDLEAWMNGVAEEARKAALPPSLRIARTNVPTVEQTWAGR<br>PPPFHDANEFKLMRYTAVATKDPIQFSDDGYVLRVHELQRPIKVFITITMYNEEGSEIKG<br>TLTGLAKGLAYMCKEYGDDFWQQVAVAIVSDGRTKASKTCLEYLKAVGAFDEEIMTVTSL<br>GVDVQMHLFESTLQLVENQNFEAYYPPLQVIYALKENNGGKLNSHLWFFNAFSEQLNPKY<br>TVLVDVGTIPAETSVFRLIRSMERNAQIGGVAGEIAVEAPNFFNPVIAAQHFEYKISNIM<br>DKSLESVFGFISVLPGAFSAYRYEAIRAVKGVGPLPEYFKSLTSTTKELGPFQGNMYLAE<br>DRILCFELLARKQRRWTMHYVKDAIARTDVPETLVDLIKQRRRWLNGSFFAGLFAIGHFG<br>RVWSQSSHSFGRKLVFTFQFVYLALQNLLSWFLLSNLFLTFYFVLTLAFTESAPALLQTM<br>LTVYLAIIGGLIVFALGNKPEPRTASFYLFSCLYMGIIMLLVTGISIYGLIGKGTSAVKD<br>PRTITGIFSNCTVSDAELAGGVITSLGLIFLSAFVHGEFGILLSFVQYFFMLPTFVNVLG<br>IYAYSNLHDLSWGTKGLESGGGHGPAKAGGGNVKDVVEQQKKIEAARQAAAREKEDVDNS<br>FRAFRSTLLLSWLTTNGIWLYVVTDYMSSGCYLKGLSYIVGFFNVVRFTGCVVFVILRMF<br>RRFGCGARASRDNYQEALPAEWQTHYNVTNRTDGRVAPPPKHAASMDPTTPHGGVYQQV
SPRG_02554	XP_012196513	*S. parasitica* CHS3	MGVPTLSKASVFRFARQPRHHGVKTRLLRSRSKTLMLGGQSTAETAQYASCNPNGDETSS<br>NSLKPLKLKDMSTDDLLRHAEQLDMHLAKIYIESQKTKALAPIVTKSIGLPQQLWEDAGV<br>APPYHSAAEFQDLRYTAVRTADPIAFSADGYSLRVHTLGKSIKVFITVTMYNEPASQLQA<br>TLTGLAGGIDYLCHQYGYDFWQEVAVVVVADGRSKTHHSVLPYLESFGAFEKNLLAQAIA<br>ASKDTHVHLFESTIQLRKTNGSFHAPMQLIFALKEHNAGKLHSHLWFFNAFSEQVDPTYT<br>ALVDVGTVPAESSVYRLIRSMERNPQIGGVAGEIAVDDPDFFNPVIAAQHFEYKIANIMD<br>ASLQSVFGFIGVLPGAFSAYRYEAIRPINGVGPLAEYFKSLTASKKELGLCVGNMYLAED<br>RILCFEILARKNCDWTMHYVKDAIAHTDVPETLVDLIKQRRRWLNGSFFAGLFAIWNFGR<br>VWTQSAHSLPRKCAFSLQFLYLAFQNVMNWFLLSNLFLTFFYILSLALYYKSIELLHVVL<br>GTYFVLVGSLIVFALGNKPGHRTAIYYRVSSYIMGTIMLCVTCISLYALLGNVQFVDPRS<br>DLPSCSVSNYELEAGAFFSLGIIFVCAFMHGEFGIVRSTVQYFFMLPTFVNVLGIYAYSN<br>LHDLSWGTKGIETSAGHNGLPTSKFGSVKDMVALHLNATSTTDVVSEADKRKGVVAAEHE<br>DVDNRFRVFRSLLLLTWLLTNGCWLYYATSFISCSCYLKYLSYIVAVFNTFRFLGGLLFL<br>SFRMARGALHCCRQGVKKTRPLRCGNPQAGDDCSPV
SPRG_19383	XP_012196264	*S. parasitica* CHS4	MTTLPERLLARVTSSMSALGGTAKLVTAQEGFRLIEQGVLAERQQHYKEAVDRFLGAAGV<br>LDAVAATEADLHVRRLLHAKASDVVAWTEGIVAWMQHRPEVRPAYPPRQSKGISMPTTTV<br>SAATLAFGMDEVERTSLHYTPVLTRSPSEFSRDGYELQVLRRHRRPRMLIVITMYNEDGS<br>EIEATLRKVGNNVAYLCRHDLPGYEGELAWQNVLVVIVSDGRAKASASTLITLREMGVYD<br>EDTLRITSAGLATSMHLFERTLLLPEAPGAKKLWTHTSETMPPLQVVFALKEENAGKLHS<br>HLWFFHGFCNQVDPTYTVLLDVGTLPTKSALYKLVSAMEVNQQVGGVCGEIAVSQPLPHL<br>TSLIISTQHYEYKISNVLDKATESCFGFISVLPGAFSAYRFKAIQGAPLDAYFKSLTTDM<br>LALGPFQGNMYLAEDRILCFELLARKNCSWTMMYVKDAIARTDVPTTLVDLMAQRRRWLN<br>GSFFAMLYTIFNWGRVYSEARHSLCRGLALLVQYTFMTVQVVFNWFLVANFYLTVYYVIF<br>YALERNALGVLDTRAFYASHGALAKGLFNVVYGLVFVVQIILGMGNRPKHVARTYRAIGA<br>YYMLLVVLTTAASVLTLVHTGAAALAPKEIALGIAVFGVYFIAAACHCELHHIVLSFVQY<br>SLLLPVMINTLTIYSFCNLQDLSWGTKGIDTSSHDTGASENGEYKDVVARQKAAEDRAKQ<br>AAATTDLVRRRFDSFRSNLLLLWLVSNAALVGGLLYGALLDVYLPCLFVAIGAFNTYRLL<br>GSLLFLLYTGRQWLLLQLCLCCGCLRRRYDRERRRGSDDRTMAILSPRDPLATI

SPRG_02074	XP_012196021	*S. parasitica* CHS5	MVSSQLSTGLPAIARRPSVRSTRVGSHVRNDNDCVTTVDAFRYIERGVRAEYDMFYSEAI<br>NCFVNAGECLLIVAEQNDDDVSQMLLAKSQEVIGWAEELSIWLENGRAGPLPSRNCRGIQ<br>IPFTKEYEGGEHYEEAAELSYTPVATVNPINFTLDGYRMQCVTRGRKPTMMLVITMYNED<br>GAELAQTLRKVCNNVKYIQKNALPGYEGDDAWQNIVVCIVSDGRTKANPSATSFLRDIGV<br>FNEDAMTIFSSGAATQMHLFERTVRLAKDPLNKQSVIMSNNSTIGADYPPLQMVYALKEH<br>NAGKLNSHLWFFNAFCNQVDPEYNILLDVGTLPTKAALYKLLATLEMKADIGGVCGEIAV<br>SRPIPNLWNFVIATQHFEYKVSNLLDKATESCFGFVSVLPGAFSAYRFAAIKGAPLQAYF<br>KSLTTDMAELGPFYGNMYLAEDRILCFELLARTNGAWKLKYIKDAVARTDVPSTLVDLMA<br>QRRRWLNGSFFAMLYSIVQWGRLYSHTNHSLFTKAGLLIQYFQLLVQLFFGWFMCGFFYL<br>SVYYVVFTTLKKSKLPFWDSEEWYDDHHSMAMSIFNIVYAFLIMVQIIFGLGNKPKHVKW<br>LYTFLSIFYAIVVITAVFFSVCSLSSHNGMSSFNIVLLAATFGVYFIAAAFHFELHHVVF<br>TFVQYLVMLPTTINILMIYAFCNIQDLSWGTKGLGDSAGHGPTKGGGQRLGSGGYSDLVA<br>QRKAAEAAARHDAVVADQVKRRFDSFRSYTLLFWLISNALLIMTCIYFVGANVFLPSLFL<br>FIALFNVTRLLGSIAFVLATGRDWLLLKLCLCSGGMAKRKQKKEKVKDQDGFGALDSSKV<br>LRHSA
SPRG_06131	XP_012200244	*S. parasitica* CHS6	MSRRNYAPAARGGNGPRPNMNPANMGPPPQMPPPQHGSGRNLRAPPPRQVQQQGSFDRDS<br>DDDMYGQHGANGVLGVPVWQDSAAYEHGSYLQENTPPSQRIPYPGPQGGGMMPPPGGMMP<br>PPQQFAPAPVRMLNAGALQNQSNQQMQVMRDSNVGQAVPASTPEAFRIIASGVTAEAEAR<br>YQSAVNDFLAGGEMLALVSEREADPHIRSLLNTKAIQVLEWSKNLHDWYQQGMRGPMPRR<br>FVGKIGVNVVNRLGACAGRIDAGSPSELRTMYYTPAANKVVSDFTKDGYRLQCIEEGRTP<br>QLMVVITMYNEDQVEMYSTLKKVANNIAHIKSQKLPGYEGDDAWKNILVVIVSDGRTKAN<br>KGTLAFLRDVGAFDEDVMNILMVGVDVMCHVFEFCVQLKKANTIEASASSSERYPPTQVV<br>FALKEHNGGKLNSHEWYFNAFAEQIQPEYTVLLDVGTMPTAKAFYLLLCAMEIDPQIGGT<br>CGEIAVDKPIPHLCNWVIAAQHFEYKISNVMDKSLESVFGFISVLPGAFSAYRYKAIRGA<br>PLEAYFKSLTTPMNELGPFQGNMYLAEDRILCFELLARRNCRWTMQYVKDAIARTDVPTD<br>LVALIGQRRRWLNGSFFALVYTILNWGRVYTESNHSYIRKFFLCIQYAYMTANVALSWVL<br>PANFFLVTYFLVIVGFKQNNWGYIPTSGIPENTKDIIVQVFSLLYGTSFLIQLVAGLGNK<br>PKHIKGVYRLTAVFYALVMLLTSVIAFGFIMKPWIDSLRSGVPFAAMVTSFEIKDIAAFV<br>ASVGVFFLASCLHCEMHHIAMSFIQYMCLLPTFVNILNTYSFCNLHDLSWGTKGLESSDG<br>HGPKAGGGGNYKDAAEAKKAEEARKKKEGEIKDKMEGDFQYFRSKLLIFWLLSQMGFAYL<br>IISFDSVNGQEGANYLKFLFYIVAGFNLFRLFGSTYFLLIEARICVEKMCIRGTMRDRLK<br>AKKKKQAIRAAQTQHV

[1]The sequences for XP_012196513, XP_012196264, XP_012196021 and XP_012200244 in NCBI do not match the sequences presented in this table as the latter were modified after experimental sequencing of the genes.

Table S2. List of primers used for PCR experiments.

Gene	
Chs1	Fwd TCCTCGTGCATGCTCTTCTC<br>Rev AAAGACGTTAAACGCCGTCG
Chs2	Fwd CACGATCACCGGGATCTTCTC<br>Rev CCAGCACGTTGACAAACGTC
Chs3	Fwd CCCACCTTTGTCAATGTGCT<br>Fwd2 CAAGAAGTCGCTGTCGTCGTC<br>Rev CTGGTCGCGTTCAAATGGAG<br>Rev2 GAAGAAGTCGGGATCATCGAC
Chs4	Fwd GACGTATACGGTGCTCTTGG<br>Fwd2 ACTGCGAGCTACATCACATC<br>Rev TGCTGCGTCGAAATGATGAG<br>Rev 2 GATGATGACACTCTTTCATGTG
Chs5	Fwd CGCTCAAGAAATCGAAATTGCC<br>Fwd2 AGATCGCATCTTGTGCTTCGAG<br>Rev AAGATCGAGAGGAAGGTGTAGAG
Chs6	Fwd TACTTTCTCGTCATTGTCGGCT<br>Rev GTACACGCCTTTGATGTGCT<br>Rev2 CATCAACGCAGCAAAAGGAC
EF	Fwd CATGATCACGGTTCGAGGC<br>Rev CGAGACTTCCTCCTTGATTTCCT
Tubulin	Fwd ACCGTATCCCAACTCAACTC<br>Rev ACTTGGGCGTCAATGGTC
Ubiquitin	Fwd CGACCTCGATCCAGTCGCTG<br>Rev CGACAAAGCAGGCTTACGACATG
18SrRNA	Fwd GCTGCCGCTGATCAAGCACT<br>Rev TCCGCTTGATGAAGCCGTCGTC
GAPDH	Fwd TCGTGGTACGACAACGAGTGG<br>Rev TGATGCTGTCGCGTCTTCTTGC
Actin	Fwd TACTCGGTCTGGATCGGTGGT<br>Rev CTTAGAAGCACTTGCGGTGGAC

Table S3. Predicted post-translational modifications and deduced molecular mass of chitin synthases from *S. parasitica*.

	**SpCHS1**	**SpCHS2**	**SpCHS3**	**SpCHS4**	**SpCHS5**	**SpCHS6**
**Molecular mass (Da)**	100272.7	106514.91	91052.99	92955.88	94779.62	108886.62
**Phosphorylation sites**	16	12	11	12	13	13
**Glycosylation sites**	1	1	1	-	-	-

Figure S1. Alignment of *S. parasitica*, *S. monoica*, *S. cerevisiae* and *N. crassa* CHS amino acid sequences. Conserved D,D,D and QXXRW motifs are marked in yellow. Conserved fungal sequences (a to h) are highlighted in boxes. Potential glycosylation and phosphorylation sites are marked in green and red, respectively. MIT domains are highlighted in blue.

```
ScCHS2      ------------------------------MTRNPFMVEPSNGSPNRRGASNLSKFYANA   30
NcCHS2      ------------------------------------------------------------   0
SpCHS3      ------------------------------------------------------------   0
SmCHS2      -MSDQLDLAARLRALREGNAAPADPE-----APPPTQPAPAP----QYHPQRLPPLYTQE   50
SpCHS2      MSDSNLDLAARLRALREGGAEPAPAP-----APTPYMHSPPS-------RTRPTPLYTQE   48
SpCHS4      ------------------------------------------------------------   0
SpCHS5      ------------------------------------------------------------   0
SpCHS6      MSRRNYAP------AARGGNGPRPNMNPANMGPPPQMPPPQHGSGRNLRAPPPRQV--QQ   52
SmCHS1      --------------------------------MPPKRPT--TDGRRAYNAGNTTV-----   21
SpCHS1      --------------------------------MPPKRPT--EASGRRYAPPAGRPS--NN   24

ScCHS2      NSNSRWANPSEESLEDSYDQSNVFQGLPASPSRAALRYSPDRRHRTQFYRDSAHNSPVAP   90
NcCHS2      -----------------------------MESRISNRLSSSATRTVRAFRNRCHARPGTP   31
SpCHS3      ------------------------MGV---------------------------------   3
SmCHS2      SLEFG--GTYATGSPVGAEAEGSYSQVPVWKDSKETRR----------------------   86
SpCHS2      SLEFG--GTYTTGSPVGAEADGVYTQVPVWKDSKEKTY----------------------   84
SpCHS4      ------------------------------------------------------------   0
SpCHS5      ------------------------------------------------------------   0
SpCHS6      QGSFD---RDSDDDMYGQHGANGVLGVPVWQDSAAYEH----------------------   87
SmCHS1      RAPTK---RTQPRGKIGSRA----SNNPSAASMQAYEF----------------------   52
SpCHS1      AANAK---PRAPRKGVSSRA----SNVPSAASS----Y----------------------   51

ScCHS2      NRYAANLQESPKRAGEAVIHL---SEGSNLYPRDN-ADLPVDPYHLSPQQQPSNNLFGSG   146
NcCHS2      GSSYGNARR-PLPSAPAPLHYNSPSRAASHYPRYH-GGYADDVTVSMGPDDDRTDIFGPE   89
SpCHS3      ----------PT-----------LS-------------------------KASVFRFARQ   17
SmCHS2      ----SYLDDEPT-----------PQPQSL----------INMANTLV-QRQASNQSFRR-   119
SpCHS2      ----GYLDDEPA-----------PQAQTL----------LNKANDLV-QRQASNKAFRR-   117
SpCHS4      ------------------------------------------------------------   0
SpCHS5      ---------------------------------------------MV-SSQ------LST   8
SpCHS6      ----GSYLQENT-----------PPSQRIPYPGPQGGGMMPPPGGMMPPPQQFAPAPVR-   131
SmCHS1      --------------------------------EYDYNSAMMPM----L-QPPKSQPTFLNN   76
SpCHS1      --------------------------------EYDYEYNMMPM----MQAPPKSQPTFLSN   76

ScCHS2      R-------------------------LYSQSS----KYTMSTTSTTAPSLAEADDEKEKY   177
NcCHS2      T-------------------------DLSETRHLNDAYGFRSSQIT---LSE--DP--HG   117
SpCHS3      --------P-RHHGVKTRLLRSRSKTLML---------------------------GGQS   41
SmCHS2      -----------QHTANFRPLPNTVEELLDGTPTYEGAFRLVQLAVQ----ME---QDGDP   161
SpCHS2      -----------QHTAAFRPLPNTVEELLDGSPTYEGAFRLVQLAVQ----ME---QDGDP   159
SpCHS4      -MTTLPERLLARVTSSMSALG-----GTAKLVTAQEGFRLIEQGVL----AE---RQQHY   47
SpCHS5      GLPAIARRPSVRSTRVGSHVR-----NDNDCVTTVDAFRYIERGVR----AE---YDMFY   56
SpCHS6      --MLNAGALQNQSNQQMQVMR-DSNVGQAVPASTPEAFRIIASGVT----AE---AEARY   181
SmCHS1      IAPISSKEASMKSSNAMQLL------LQSTSFTIDDAFRAIERAIQ----AE---NEGRY   123
SpCHS1      IAPISAKEASMKGSNAMQLL------LQGTSFTIDDAFRAIERAIQ----AE---NEGRF   123
```

```
ScCHS2          LTSTTSYDDQ---STIFSADT-----------FNETKFELNHPTRQQYVRRANSESKRRM    223
NcCHS2          THARSRYDDEDDVSTTYSSNTGT-------SASGVDKFEHYGPIPE--------EGKHER    162
SpCHS3          TAETAQYASCNPNGDETSSNSLKPLKLKDM--STDDLLRHAEQLD-MHLAKIYIESQ-KT     97
SmCHS2          GAAINLYVDAGTTLVEVGKREVDPLLQKGIQQKAFELLQRAEELG-TWMNTVAEEAR-KA    219
SpCHS2          QAAINLYADAGATLVEVGRKEVDPLLQKGIRQKAQELLQRAEDLE-AWMNGVAEEAR-KA    217
SpCHS4          KEAVDRFLGAAGVLDAVAATEADLHVRRLLHAKASDVVAWTEGIV-AWMQHRPEV-----    101
SpCHS5          SEAINCFVNAGECLLIVA-EQNDDDVSQMLLAKSQEVIGWAEELS-IWLENGRA------    108
SpCHS6          QSAVNDFLAGGEMLALVSEREADPHIRSLLNTKAIQVLEWSKNLH-DWYQQGMR------    234
SmCHS1          REALKHFLDGGEMIVTAAEKEASQKVRNLLLHKGKEVLEWAEHLA-EWIERYNT------    176
SpCHS1          REALKHFLDGGEMIVTAAEKEASQKVRNLLLHKGKEVLEWAEHLA-EWIERYNT------    176
                      :          .                   .

ScCHS2          VSDLPPPSK------KKALLKLDNPIPKGLLDTLPRRNSPEFTEMRYTACT-VEPDDFLR    276
NcCHS2          RGVRPPQMSRKEVQLINGELVLECKIPTILYSFLPRRDEVEFTHMRYTAVT-CDPDDFVA    221
SpCHS3          -KALAP-IVTKSIGLPQQLWE------DA-GVAPPYHSAAEFQDLRYTAVRTADPIAFSA    148
SmCHS2          --ALPPQLKIARTNVPTVEQA------WK-GRTPPFHDADEFRLMRYTAVATKDPIQFSN    270
SpCHS2          --ALPPSLRIARTNVPTVEQT------WA-GRPPPFHDANEFKLMRYTAVATKDPIQFSD    268
SpCHS4          -RPAYPPRQSKGISMPTTTVS--------AATLAFGMDEVERTSLHYTPVLTRSPSEFSR    152
SpCHS5          --GPLPSRNCRGIQIPFTKEY--------EG----GEHYEEAAELSYTPVATVNPINFTL    154
SpCHS6          --GPMPRRFVGKIGVNVVNRL------GACAGRIDAGSPSELRTMYYTPAANKVVSDFTK    286
SmCHS1          --HSAPVRVAKPMAVEVTYDR------TMNSPD---LDETEARTMFYTPVCCT-PQAFTE    224
SpCHS1          --STPPVRIAKPMAVEVTYDR------TMNSPD---LDETEARMMFYTPVCSG-PKAFTE    224
                     *                                  *   : **         *

                                        a
ScCHS2          EGYTLRF--AEMNRECQIAICITMYNEDKYSLARTIHSIMKNVAHLCKREKSHVWGPNGW    334
NcCHS2          RGYKLRQNIGRTARETELFICVTMYNEDEFGFTRTMHAVMKNISHFCSRNKSRTWGADGW    281
SpCHS3          DGYSLRV--HTLGKSIKVFITVTMYNEPASQLQATLTGLAGGIDYLCHQ-----YGYDFW    201
SmCHS2          DGYVLRV--HQLHRRIKVFITITMYNEEGSEILGTLTGLAKGLGYMCKE-----YGQDFW    323
SpCHS2          DGYVLRV--HELQRPIKVFITITMYNEEGSEIKGTLTGLAKGLAYMCKE-----YGDDFW    321
SpCHS4          DGYELQV--LRRHRRPRMLIVITMYNEDGSEIEATLRKVGNNVAYLCRHDLPGYEGELAW    210
SpCHS5          DGYRMQC--VTRGRKPTMMLVITMYNEDGAELAQTLRKVCNNVKYIQKNALPGYEGDDAW    212
SpCHS6          DGYRLQC--IEEGRTPQLMVVITMYNEDQVEMYSTLKKVANNIAHIKSQKLPGYEGDDAW    344
SmCHS1          TGYRLQC--IQSGRRPRLMVVITMYNEDENELRSTLRKVCNNVLYLKQQSLPGYEGDDAW    282
SpCHS1          TGYRLQC--IQSGRRPRLMVVITMYNEDENELRSTLRKVCNNVLYLKQHSLPGYEGDDAW    282
                 ** ::       :   : : :*****    :  *:  :  .: ::  .      *   *

                         b
ScCHS2          KKVSVILISDGRAKVNQGSLDYLAALGVYQEDMAKAS-VNGDPVKAHIFELTTQVSINAD    393
NcCHS2          QKIVVCVVSDGREIIHPRTLDALAAMGVYQHGIAKNF-VNQKAVQAHVYEYTTQVSLDSD    340
SpCHS3          QEVAVVVVADGRSKTHHSVLPYLESFGAFEKNLLAQAIAASKDTHVHLFESTIQLRKT--    259
SmCHS2          QEVAVAIVSDGRTKASKTCLEYLNGLGAFDEEIMTVT-SLGVDVQMHLFESTLQLVENQT    382
SpCHS2          QQVAVAIVSDGRTKASKTCLEYLKAVGAFDEEIMTVT-SLGVDVQMHLFESTLQLVENQN    380
SpCHS4          QNVLVVIVSDGRAKASASTLITLREMGVYDEDTLRIT-SAGLATSMHLFERTLLLPEAPG    269
SpCHS5          QNIVVCIVSDGRTKANPSATSFLRDIGVFNEDAMTIF-SSGAATQMHLFERTVRLAKDPL    271
SpCHS6          KNILVVIVSDGRTKANKGTLAFLRDVGAFDEDVMNIL-MVGVDVMCHVFEFCVQLKKANT    403
SmCHS1          KQVLVVIVSDGRTKANKGTLEWLSNVGLYDEDVMNIT-STGVKVQCHLFEHSLQMTKEN-    340
SpCHS1          KQVLVVVVSDGRTKANKGTLEWLANVGLYDEDVMNIT-STGVKVQCHLFEHSLQMTKEN-    340
                ::: * :::***          *  .* ::.            .  *::*    :

                                                                         c
ScCHS2          LD-------YV--SKDIVPVQLVFCLKEENKKKINSHRWLFNAFCPVLQPTVVTLVDVGT    444
NcCHS2          LM-------FKGAEKGIVPCQMIFCLKEKNQKKLNSHRWFFNAFGKALNPNVCILLDVGT    393
SpCHS3          N------------GSFHAPMQLIFALKEHNAGKLHSHLWFFNAFSEQVDPTYTALVDVGT    307
SmCHS2          F------------ENYFPPLQVIYALKENNGGKLNSHLWFFNAFSEQLNPKYTVLVDVGT    430
SpCHS2          F------------EAYYPPLQVIYALKENNGGKLNSHLWFFNAFSEQLNPKYTVLVDVGT    428
SpCHS4          AKKLWT-----HTSETMPPLQVVFALKEENAGKLHSHLWFFHGFCNQVDPTYTVLLDVGT    324
SpCHS5          NKQSVIMSNNSTIGADYPPLQMVYALKEHNAGKLNSHLWFFNAFCNQVDPEYNILLDVGT    331
SpCHS6          I------EASASSSERYPPTQVVFALKEHNGGKLNSHEWYFNAFAEQIQPEYTVLLDVGT    457
SmCHS1          -------------SIRFPPLQVTFALKEHNAGKLDSHLWYFDAFAEQVMPDYTVLLDVGT    387
SpCHS1          -------------SIRFPPLQ------------LDSHLWYFDAFAEQIMPDYTVLLDVGT    375
                                  * *            :.** * *..*   : *    *:****
```

```
                                                                      d
ScCHS2     RLNNTAIYRLWKVFDMDSNVAGAAGQIKTMKGKWGLKLFNPLVASQNFEYKISNILDKPL    504
NcCHS2     RPGGTSLYHLWKAFDTDSNVAGACGEIKAMKGRFGGNLLNPLVASQNFEYKMSNILDKPL    453
SpCHS3     VPAESSVYRLIRSMERNPQIGGVAGEIAVDD----PDFFNPVIAAQHFEYKIANIMDASL    363
SmCHS2     IPAETSVFRLIRSMERNYQIGGVAGEIAVEA----PNYFNPVIAAQHFEYKISNIMDKSL    486
SpCHS2     IPAETSVFRLIRSMERNAQIGGVAGEIAVEA----PNFFNPVIAAQHFEYKISNIMDKSL    484
SpCHS4     LPTKSALYKLVSAMEVNQQVGGVCGEIAVSQP--LPHLTSLIISTQHYEYKISNVLDKAT    382
SpCHS5     LPTKAALYKLLATLEMKADIGGVCGEIAVSRP--IPNLWNFVIATQHFEYKVSNLLDKAT    389
SpCHS6     MPTAKAFYLLLCAMEIDPQIGGTCGEIAVDKP--IPHLCNWVIAAQHFEYKISNVMDKSL    515
SmCHS1     MPTKSSFYKLLTALEINAQIGGVCGEIAVDKP--LPNMCNWVIAAQHFEYKISNILDKSL    445
SpCHS1     MPTKSSFYKLLTALEINAQIGGVCGEIAVDKP--LPNMCNWVIAAQHFEYKISNILDKSL    433
                :.: *   :: . ::.*..*:* .       .  . ::::*::***::*::*

                    e                                             f
ScCHS2     ESVFGYISVLPGALSAYRYRALKNHEDGTGPLRSYFLGETQEGRDHDVFTANMYLAEDRI    564
NcCHS2     ESVFGYITVLPGALSAYRYHALQNDETGHGPLSQYFKGETLHGQHADVFTANMYLAEDRI    513
SpCHS3     QSVFGFIGVLPGAFSAYRYEAIRP-INGVGPLAEYFKSLTASKKELGLCVGNMYLAEDRI    422
SmCHS2     ESVFGFISVLPGAFSAYRYEAIRA-VKGVGPLPEYFKSLTSTTKELGPFQGNMYLAEDRI    545
SpCHS2     ESVFGFISVLPGAFSAYRYEAIRA-VKGVGPLPEYFKSLTSTTKELGPFQGNMYLAEDRI    543
SpCHS4     ESCFGFISVLPGAFSAYRFKAIQG-----APLDAYFKSLTTDMLALGPFQGNMYLAEDRI    437
SpCHS5     ESCFGFVSVLPGAFSAYRFAAIKG-----APLQAYFKSLTTDMAELGPFYGNMYLAEDRI    444
SpCHS6     ESVFGFISVLPGAFSAYRYKAIRG-----APLEAYFKSLTTPMNELGPFQGNMYLAEDRI    570
SmCHS1     ESCFGFISVLPGAFSAYRYKAIRG-----APLQAYFKSLTTPMAELGPFAGNMYLAEDRI    500
SpCHS1     ESCFGFISVLPGAFSAYRYKAIRG-----APLQAYFKSLTTDMAELGPFAGNMYLAEDRI    488
           :* **:: *****:****: *::       .**  ** . *      .   .*********
                                             g
ScCHS2     LCWELVAKRDAKWVLKYVKEATGETDVPEDVSEFISQRRRWLNGAMFAAIYAQLHFYQIW    624
NcCHS2     LCWELVAKRGERWVLKYVKGCTGETDVPDTVPEFVSQRRRWLNGAFFAAVYSLVHFRQIW    573
SpCHS3     LCFEILARKNCDWTMHYVKDAIAHTDVPETLVDLIKQRRRWLNGSFFAGLFAIWNFGRVW    482
SmCHS2     LCFELLARKHKQWTMHYVKDAIARTDVPETLVDLIKQRRRWLNGSFFAGLFAIGHFGRVW    605
SpCHS2     LCFELLARKQRRWTMHYVKDAIARTDVPETLVDLIKQRRRWLNGSFFAGLFAIGHFGRVW    603
SpCHS4     LCFELLARKNCSWTMMYVKDAIARTDVPTTLVDLMAQRRRWLNGSFFAMLYTIFNWGRVY    497
SpCHS5     LCFELLARTNGAWKLKYIKDAVARTDVPSTLVDLMAQRRRWLNGSFFAMLYSIVQWGRLY    504
SpCHS6     LCFELLARRNCRWTMQYVKDAIARTDVPTDLVALIGQRRRWLNGSFFALVYTILNWGRVY    630
SmCHS1     LCFELLARKDCNWTMHYVKDAIARTDVPTNLIDLVGQRRRWLNGSFFATLFAIWNWGRVY    560
SpCHS1     LCFELLARKDCNWTMHYVKDAIARTDVPTNLIDLVGQRRRWLNGSFFATLFAIWNWGRVY    548
           **:*::*:    * : *:* . ..****  :  :: *******::** :::  :: :::

ScCHS2     K-TKHSVVRKFFLHVEFLYQFIQMLFSWFSIANFVLTFYYLAGSMNL-V---I-----KH    674
NcCHS2     K-TDHTFMRKALLHVEFLYHLLQLLFTYFSLANFYLAFYFIAGGLAD-P---HVDPFNSD    628
SpCHS3     TQSAHSLPRKCAFSLQFLYLAFQNVMNWFLLSNLFLTFFYILSLALYYKSIELL------    536
SmCHS2     SQSSHTMSRKLVFTFQFFYLALQNLLSWFLLSNLFLTFYFVLTLAFTDSAPALL------    659
SpCHS2     SQSSHSFGRKLVFTFQFVYLALQNLLSWFLLSNLFLTFYFVLTLAFTESAPALL------    657
SpCHS4     SEARHSLCRGLALLVQYTFMTVQVVFNWFLVANFYLTVYYVIFYALERNALGVLDTRAFY    557
SpCHS5     SHTNHSLFTKAGLLIQYFQLLVQLFFGWFMCGFFYLSVYYVVFTTLKKSKLPFWDSEEWY    564
SpCHS6     TESNHSYIRKFFLCIQYAYMTANVALSWVLPANFFLVTYFLVIVGFKQNNWGYIPTSGIP    690
SmCHS1     TESNHSFTRKMALLVQYVYNVLQVIFSWFLPANFYLALYFVIFQGFKDNRWNFIDTSKYP    620
SpCHS1     TESNHSLTRKLALLVHALLGV--------SAANFYLALYFVIFQGFRDNRWNFIDTSEYP    600
           . : *:       : ..                . : *  :::

ScCHS2     ---GEALFIFFKYLIFCDLASLFIISMGNRPQGAKHLFITSMVIL-SICATYSLICGFVF    730
NcCHS2     GHVARIIFNILRYVCVLLICTQFILSLGNRPQGAKRMYLASMIIY-AVIMVYTTFATIFI    687
SpCHS3     ---HVV-----LGTYFVLVGSLIVFALGNKPGHRTAIYYRVSSYIMGTIMLCVTCISLYA    588
SmCHS2     ---QAM-----LTLYLAIVGGLIVFALGNKPEPRTASFYLFSCLYMGIIMMLVTGISIYG    711
SpCHS2     ---QTM-----LTVYLAIIGGLIVFALGNKPEPRTASFYLFSCLYMGIIMLLVTGISIYG    709
SpCHS4     ASHGALAKGLFNVVYGLVFVVQIILGMGNRPKHVARTYRAIGAYY-MLLVVLTTAASVLT    616
SpCHS5     DDHHSMAMSIFNIVYAFLIMVQIIFGLGNKPKHVKWLYTFLSIFY-AIVVITAVFFSVCS    623
SpCHS6     ENTKDIIVQVFSLLYGTSFLIQLVAGLGNKPKHIKGVYRLTAVFY-ALVMLLTSVIAFGF    749
SmCHS1     ALLLDGLPTAFNVFYAVTVFTQVTIGLGNKPKHVKGTHYLISVLF-GILMLIASTIAIVI    679
SpCHS1     QWVLDGLPTAFNVFYAVTVFTQVTIGLGNKPKHVKGTHYLISVLF-GLLMLLASGVAIVI    659
                             .   .  .:**:*      .                   .
```

```
ScCHS2      AFKSLASG-----------TESHKIFVDIVISLLSTYGLYFFSSLMYLDPWHMFTSSIQY    779
NcCHS2      VVRQIQPSQKSDDKPD--LELGNNVFTNLIVSVASTLGLYFVMSFLYLDPWHMFTSAIQY    745
SpCHS3      LLGNVQF--VDPR---SDLPSCSVSNYELEAGAFFSLGIIFVCAFMHGEF-GIVRSTVQY    642
SmCHS2      LVGKGTSAVKDPRVITGALGNCTVSEGELVGGVVTSLGLIFLSAFVHGEF-SILLSVIQY    770
SpCHS2      LIGKGTSAVKDPRTITGIFSNCTVSDAELAGGVITSLGLIFLSAFVHGEF-GILLSFVQY    768
SpCHS4      LVHTGA-------------AA---LAPKEIALGIAVFGVYFIAAACHCELHHIVLSFVQY    660
SpCHS5      LS--SH-------------NG---MSSFNIVLLAATFGVYFIAAAFHFELHHVVFTFVQY    665
SpCHS6      IMKPWIDSLRSGVPFAAMVTS---FEIKDIAAFVASVGVFFLASCLHCEMHHIAMSFIQY    806
SmCHS1      FVTAH-------------------KTVEAIILAVLILGTFFIGSAMHCEVHHIVLTFVQY    720
SpCHS1      FITSS-------------------KDAMAIVLAVLILGTFFIGSALHCEVHHIVLTFVQY    700
                                                 *  *. :  : :   :  : :**

                                     h
ScCHS2      FLTLPAFTCTLQIFAFCNTHDVSWGTKGSTQESKQLSKAI---VVQGPDGKQIVET--DW    834
NcCHS2      FVLLPSYICTLQIYAFCNTHDVTWGTKGDNVMRTDLGGAI---VKGSTVELEMPSDQLDI    802
SpCHS3      FFMLPTFVNVLGIYAYSNLHDISWGTKGIETSAGHNGLPTS----KFGSVKDMVALHLNA    698
SmCHS2      FFMLPTFVNVLGIYAYSNLHDISWGTKGLESGGGHGPTK-T----GGGNVKDVVEQQK--    823
SpCHS2      FFMLPTFVNVLGIYAYSNLHDISWGTKGLESGGGHGPAK-A----GGGNVKDVVEQQK--    821
SpCHS4      SLLLPVMINTLTIYSFCNLQDISWGTKGIDTSSHDTG------ASENGEYKDVVARQK--    712
SpCHS5      LVMLPTTINILMIYAFCNIQDISWGTKGLGDSAGHGPTKGGGQRLGSGGYSDLVAQRK--    723
SpCHS6      MCLLPTFVNILNTYSFCNLHDISWGTKGLESSDGHGPKAGG-----GGNYKDAAEAKK--    859
SmCHS1      TALMPSFVNILMVYSFCNLHDISWGTKGIDTGHEHK-SDGA-----VGQYKDIVARQK--    772
SpCHS1      TALMPSFVNILMVYSFCNLHDISWGTKGIDTGHEAH-KTEA-----VGQYKDIVARQK--    752
               :*      *  :::.* :*::*****                          :

ScCHS2      PQEVDKKFLEIKSRLKEPEFEESSGNEKQSKNDYYRDIRTRIVMIWMLSNLILIMSIIQV    894
NcCHS2      DSGYDECLRNLRDRVMVP---CRSLSEDQLQQDYYKSVRTYMVVSWMVANATLAMAVSEA    859
SpCHS3      TSTTDVV-SEADKR------KGVVAAEHEDVDNRFRVFRSLLLLTWLLTNGCWLYYA---    748
SmCHS2      ---------KLEAQ------RQAAAKEKEDVDNSFRAFRSTLLLSWLTTNGIWLYVV---    865
SpCHS2      ---------KIEAA------RQAAAREKEDVDNSFRAFRSTLLLSWLTTNGIWLYVV---    863
SpCHS4      ---------AAEDR------AKQAAATTDLVRRRFDSFRSNLLLLWLVSNAALVGGL---    754
SpCHS5      ---------AAEAA------ARHDAVVADQVKRRFDSFRSYTLLFWLISNALLIMTC---    765
SpCHS6      ---------AEEAR------KKKEGEIKDKMEGDFQYFRSKLLIFWLLSQMGFAYLIISF    904
SmCHS1      ---------ALEAK------KAEDARNQDELKKRFDSFRSNLLLIWVMSNMAMVVIC---    814
SpCHS1      ---------ALEAK------KAQDARNQDELKKRFDSFRSNLLLVWVMSNMSMVIIC---    794
                                       :     :   .*:  :: *: ::

ScCHS2      FTPQD-TDNGYLIFILWSVAALAAFRVVGSMAFLFMKYLRIIVSYRNKV-EG---SGSWE    949
NcCHS2      YGDSEIGDNFYLRFILWAVAALALFRALGSTTFAAINLVSALVEGRVRL-RLNMKGFRWI    918
SpCHS3      --TSFISCSCYLKYLSYIVAVFNTFRFLGGLLFLSFRMAR---GALHCCRQGVKK-TRPL    802
SmCHS2      --TDYMSSGCYLKGLSFVVGFFNVIRFTGCVVFIILRIFR--RFGLNCCAMGATH-DTYE    920
SpCHS2      --TDYMSSGCYLKGLSYIVGFFNVVRFTGCVVFVILRMFR--RFG---CGARASR-DNYQ    915
SpCHS4      --LYGALLDVYLPCLFVAIGAFNTYRLLGSLLFLLYTGRQWLLLQL-CLCCGCLR-RRYD    810
SpCHS5      --IYFVGANVFLPSLFLFIALFNVTRLLGSIAFVLATGRDWLLLKL-CLCSGGMA-KRKQ    821
SpCHS6      DSVNGQEGANYLKFLFYIVAGFNLFRLFGSTYFLLIEARICV--EK-MCIRGTMR-DRLK    960
SmCHS1      --VNTIGADSYLPFLYAFVAAFNGIRLLGCIGYLLYYARQFLLFNT-LSATGVLH-KRHE    870
SpCHS1      --VNTVGADSFLPFLYAFVAAFNGIRLLGCIGYLIYYARQFLLFNT-LSATGVLH-KRHE    850
                      :*  :   :. :    *   *    :

ScCHS2      VS-KL---DLPNVFHK-KG--------------------------   963
NcCHS2      KE-KWGDADVKGKFEG-LGDR------ARGLAKR-----------   944
SpCHS3      RCGNPQ-----------AGDD---------CSPV-----------   816
SmCHS2      RNLPPDWQTHYNVQNQADGRVVVA--RAESINPATPRGGAYQQV-   962
SpCHS2      EALPAEWQTHYNVTNRTDGRVAPPPKHAASMDPTTPHGGVYQQV-   959
SpCHS4      RERRRGSDDRTMAILS----P---RDPLATI--------------   834
SpCHS5      KKEKVKDQDGFGA-LD----S---SKVLRHSA-------------   845
SpCHS6      AKKKKQAIRAAQTQHV-----------------------------   976
SmCHS1      ARKHKKAEEDPDPIDFEMGTF---QNDLPD-V-AVPIQAPYNRMR   910
SpCHS1      ARKHKKA-EDPDPIDMELGTF---NEPATSEIGAPMMQAPYNRMR   891
```
